# Supplementary material for: Conditioned Medium of Mesenchymal Stromal Cells Loaded with Paclitaxel Is Effective in Preclinical Models of Triple-Negative Breast Cancer (TNBC)
Source: Int J Mol Sci. 2023 Mar 20;24(6):5864. doi: 10.3390/ijms24065864 (PMC10058623; doi:10.3390/ijms24065864)
Supplement: Supplementary file 1 [file ijms-24-05864-s001.zip › ijms-2200007-supplementary.pdf]

Supplementary methods

Trypan Blue assay

BT549 cells were seeded into 96-well plates (5000 cells/well or 3000 cells/well for reading after 72h or 144h respectively) and incubated for 24 hours. Growth medium was then replaced with conditioned medium (MSC-CM CTRL and MSC-CM PTX 24h / 48h) at progressively lower sequential concentrations. The CM were diluted at the same concentration. After 72h incubation, cells were mixed 1:1 with 0.4% Trypan Blue (Thermo Fisher Scientific, Waltham, 140 MA, USA). Cells permeable to Trypan Blue were counted as dead. Counts were performed by using a Bürker chamber under an inverted microscope. In parallel, cell viability was determined by CellTiter-AQueous reagent (Promega) as described in the Materials and Methods section.

KI-67 signal detection

Cells, seeded on glass coverslips, were cultured for 48 or 72 hours in DMEM or MSC conditioned medium as previously described. For fixation, cells were washed twice with PBS and then incubated for 10 minutes at room temperature in 4% paraformaldehyde in 0.12M sodium phosphate buffer, pH 7.4, then left in PBS overnight at 4 °C. Cells were incubated for 2 hours at room temperature with anti-Ki-67 (NB500-170, Novus Biologicals) primary antibody (1:100 dilution in GDB buffer [0.02M sodium phosphate buffer, pH 7.4, containing 0.45M NaCl, 0.2% (w/v) bovine gelatine, 0.2% Triton-X100]), followed by staining with Alexa 488-conjugated secondary antibody and anti-phalloidin Alexa 568-conjugated (1:100 and 1:400 dilution in GDB buffer) (Thermo Fisher Scientific) for 1 hour. After two washes with PBS and staining with Hoechst 33342 (Thermo Fisher Scientific), coverslips were mounted on glass slides with a 90% (v/v) glycerol/PBS solution.

Confocal images acquisition and analysis

Images were acquired using Zeiss LSM 710 confocal laser-scanning microscope (Zeiss) using a 63x, 1.4 N/A oil-immersion objective. Laser intensities and acquisition parameters were held constant throughout each experiment.

Confocal microscopy fields were analyzed using specific homemade-designed macro with ImageJ (https://imagej.nih.gov/ij/) software. In detail Ki-67 signal intensity was analyzed measuring the ID in nuclear compartment and normalized over the control cells cultured in DMEM. All the data obtained derived from at least 5 fields per experimental condition (at least 80 cells each).

Supplementary Figures

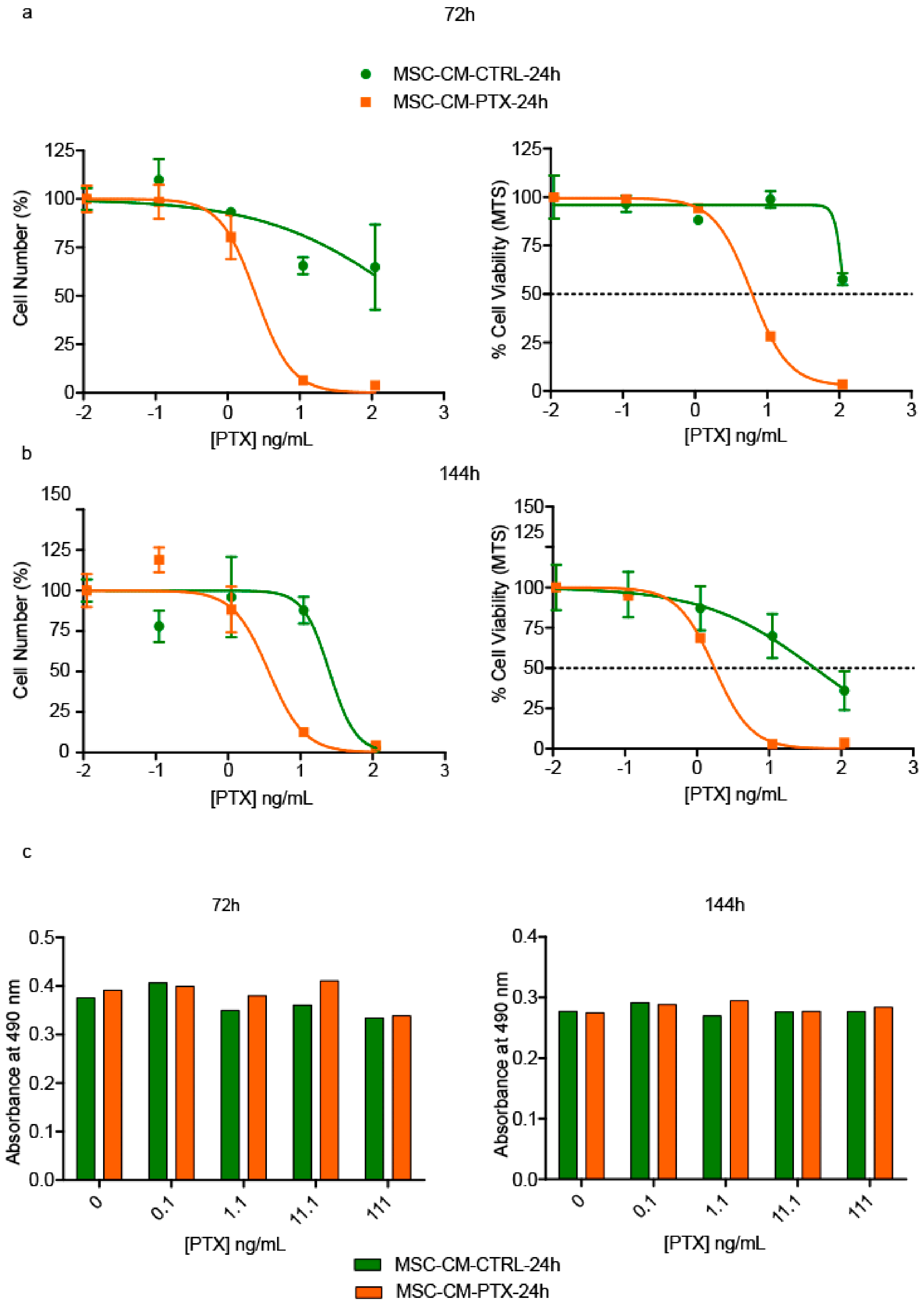

Figure S1. Cells treated with MSC-CM-PTX show a decrease in cell number associated with concurrent cell death. (**a-b**) Trypan blue count (%) for BT549 cells treated with MSC-CM-PTX (111 ng/mL, 11 ng/mL, 1.1 ng/mL 0.1 ng/mL and 0 ng/mL (DMEM) and MSC-CM-CTRL with the same dilutions is shown on the left at 72h (**a**) and at 144h (**b**). On the right, MTS viability assays performed in parallel. Values are presented as Mean ± SD. (**c**) Control of PTX-MTS cross reaction: the histograms report the absorbance at 490 nm of MSC-CM-PTX and MSC-CM-CTRL media at different concentrations mixed with the MTS solution; the absorbance is equal to DMEM, both at 72h and 144h, indicating there is no interference of PTX with MTS signal.

a

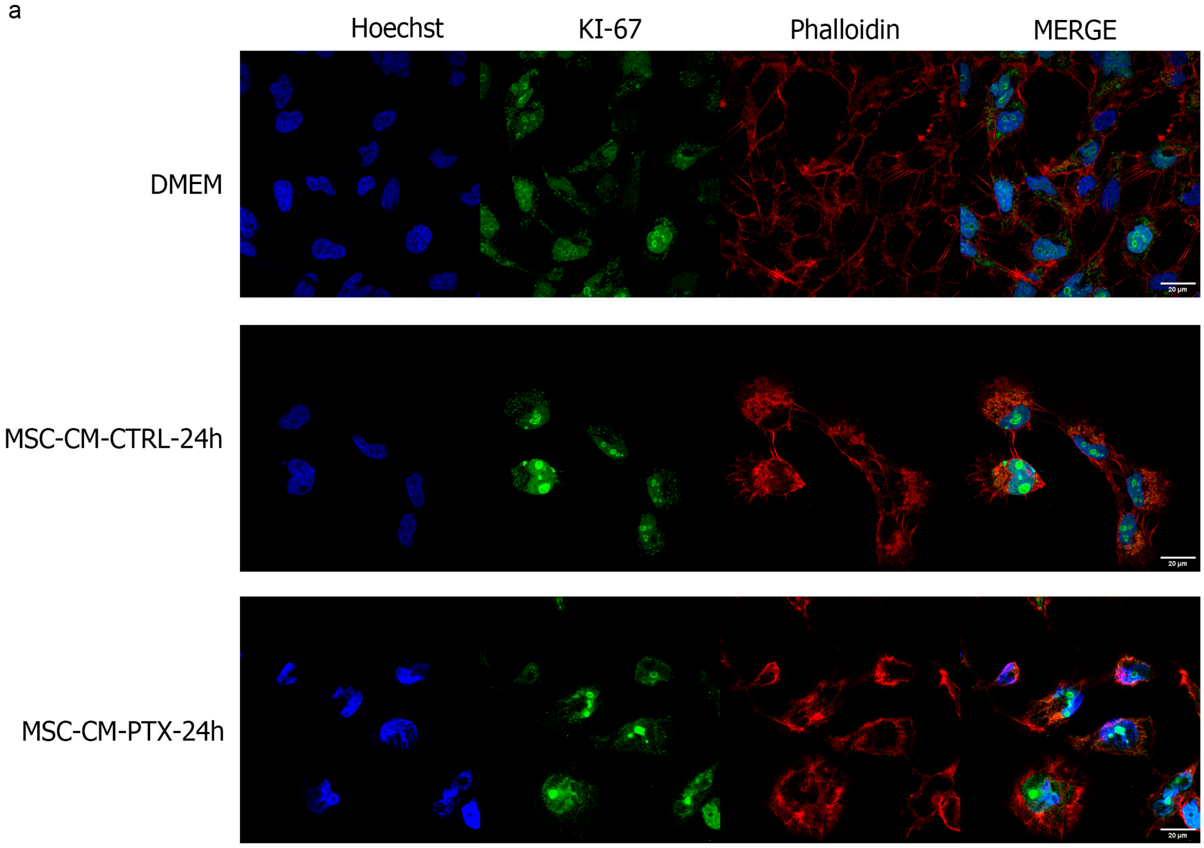

b

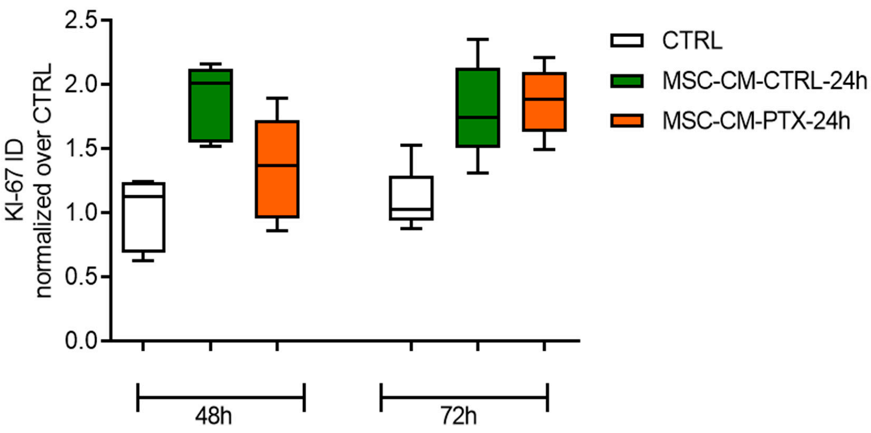

Figure S2. Ki-67 intensity density normalized versus CTRL. (**a**) Representative images of BT549 cells treated with CTRL (DMEM), MSC-CM-CTRL and MSC-CM-PTX 1.11 ng/mL at the same dilution, at 48h (nuclei are stained in blue with Hoechst, Ki-67 in green, Phalloidin in red). (**b**) Ki-67 signal intensity was analyzed measuring the ID in nuclear compartment and normalized over the control cells cultured in DMEM. All the data obtained derived from at least 5 fields per experimental condition (at least 80 cells each).
